# Spheroscope: A custom-made miniaturized microscope for tracking tumour spheroids in microfluidic devices

**DOI:** 10.1038/s41598-020-59673-1

**Published:** 2020-02-17

**Authors:** A. Rodríguez-Pena, J. Uranga-Solchaga, C. Ortiz-de-Solórzano, I. Cortés-Domínguez

**Affiliations:** 1 IDISNA, Ciberonc and Solid Tumours and Biomarkers Program, Center for Applied Medical Research, University of Navarra, 31008 Pamplona, Spain; 2 USCAL, S.L. Ingeniería Mecatrónica + Dirección, Pol. Industrial Arazuri-Orcoyen, Calle C - No1, 31160 Orcoyen, Spain

**Keywords:** Cancer imaging, Integrated optics

## Abstract

3D cell culture models consisting of self-assembled tumour cells in suspension, commonly known as tumour spheroids, are becoming mainstream for high-throughput anticancer drug screening. A usual measurable outcome of screening studies is the growth rate of the spheroids in response to treatment. This is commonly quantified on images obtained using complex, expensive, optical microscopy systems, equipped with high-quality optics and customized electronics. Here we present a novel, portable, miniaturized microscope made of low-cost, mass-producible parts, which produces both fluorescence and phase-gradient contrast images. Since phase-gradient contrast imaging is based on oblique illumination, epi-illumination is used for both modalities, thus simplifying the design of the system. We describe the system, characterize its performance on synthetic samples and show proof-of-principle applications of the system consisting in imaging and monitoring the formation and growth of lung and pancreas cancer tumour spheroids within custom made microfluidic devices.

## Introduction

Since the advent of the first scientific microscopes in the XVI century, microscopes have evolved thanks to the development of increasingly corrected optical elements and improved illumination strategies, to provide brighter, clearer, artefact-free images. During the last century, the use of image acquisition devices and integrated control electronics, started the digital era of microscopy, allowing the automation of microscope systems, and introducing the use of digital signal processing for the analysis of electronically acquired images. More recently, novel engineering solutions have been proposed that artificially “break” the diffraction limit imposed by the physics of the image formation process, increasing the resolving power of the microscope to the order of a few tens of nanometers^1,2^. In parallel, following the “do it yourself” (DIY) philosophy^3^, new home-made microscopy systems have been developed, custom-made for specific applications. Common to these DIY microscopy systems are features such as portability, low-cost and customization for a particular application. Some of these systems have been developed in a community collaborative fashion, by allowing multiple technologists to participate in the design, and providing public, detailed step-by-step building instructions and a significant level of technical support. Examples of these are the Miniscope^4^ (http://miniscope.org), a miniaturized fluorescent microscope for the study of the activity of the brain in free-moving mice; the Wireless platform^5^, a wireless capable miniaturized microscope; or the OpenSpim^6^ (https://openspim.org/), a light sheet microscope for mesoscopic observation of 3D samples. Moreover, other laboratories have developed home-made devices displaying a wide variety of technologies such as CARS^7^, Fourier ptychographic microscopy^8^ or single optical fibre microscopy^9^.

Within the field of cancer biology, there is growing interest in the use of 3D cell cultures^10^ designed to bridge the physiological gap existing between the *in vitro* and *in vivo* traditional models. Spheroids -i.e. 3D aggregates of cells in suspension- have been introduced in the last decade to model tissues^11,12^ and study diseases^13,14^, including cancer. Several strategies can be used to generate these spheroids, including the use of microfluidic technologies. Among other virtues, microfluidic platforms offer high control and easy manipulation of the spheroid microenvironment while reducing, due to their microscopic size and experimental integration, the amount of reagents required, which in turn reduces the cost of the experiments^15,16^.

General purpose microscopes, equipped with high quality optics and electronics, are commonly used to track *in vivo* the growth of tumour spheroids. These systems are therefore underused for what can be considered a relatively simple imaging task. Alternatively, a few commercial, expensive dedicated systems exist, mainly designed to track 3D cultures on relatively large support systems, e.g. multiple-well plates. Here we present the Spheroscope, a novel miniaturized, inverted microscope designed for imaging and tracking the formation and growth of tumour spheroids in microfluidic platforms, both in epi-fluorescence^17^ and epi-illumination phase-gradient contrast imaging based on oblique back-scattered illumination (OBM)^18^. The Spheroscope, originally designed by us following the DIY principles^19^, is built of low-cost gradient refractive index (GRIN) lenses, miniaturized optical elements and consumable electronics, but provides a resolution, sensitivity and aberration-free field of view that make it suitable for long term imaging of live cellular samples at a low-intermediate level of resolution. Here we do a detailed description of the elements of the system, quantitatively characterize its performance and show, as proof-of-principle, its use to monitor in 2D images the formation and growth of 3D tumour spheroids created from lung and pancreatic cancer cell lines within customized microfluidic devices.

## Results

### Spheroscope implementation and characterization

**Figure 1:**
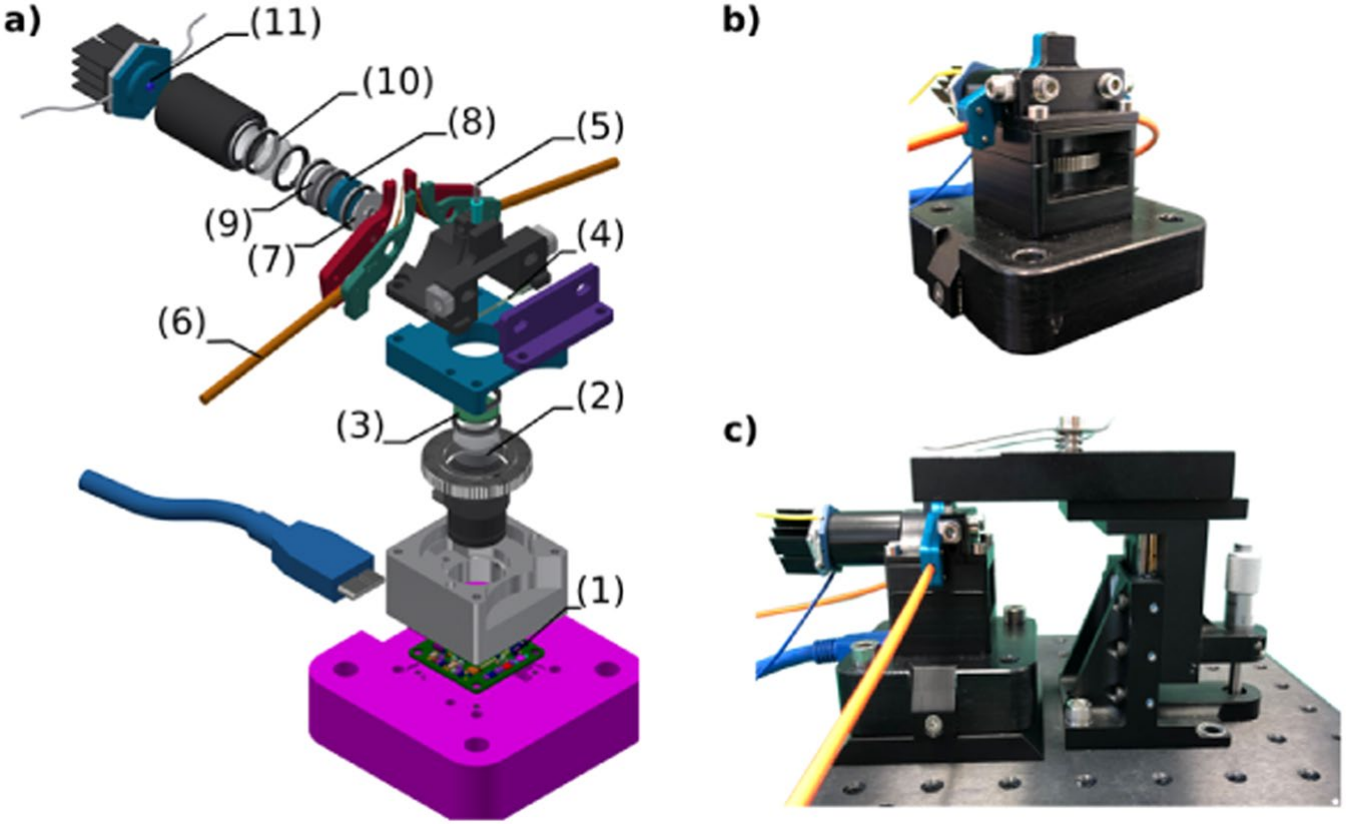
Spheroscope design and implementation. (**a**) Expanded-view of the microscope design with the main elements highlighted: (1) CMOS sensor; (2) achromatic lens; (3) emision filter; (4) dichroic mirror; (5) objective GRIN lens; (6) fiber optic cable; (7) diaphragm; (8) excitation filter; (9) diffuser lens; (10) condenser lens and (11) blue LED. (**b**) Implentation of the Spheroscope prototype. (**c**) Spheroscope with a micro-positioner stage.

The optical design of the Spheroscope (Fig. 1) is inspired on the fluorescence and OBM designs proposed, respectively by Gosh *et al*. (2011)^4^ and Ford *et al*. (2012)^20^, harmonized and combined into a simple, integrated design, and adapted to provide the working distance and field of view required for our particular application. The Spheroscope housing body that encapsulates all the optical and electronic elements is made of aluminium, with a size of 7 × 9 × 7.2 cm and a weight of 375gr. The approximate cost of its components, bought as single units, is 2500€, amount that could be considerably reduced if the components were purchased in large numbers for mass production.

**Figure 2:**
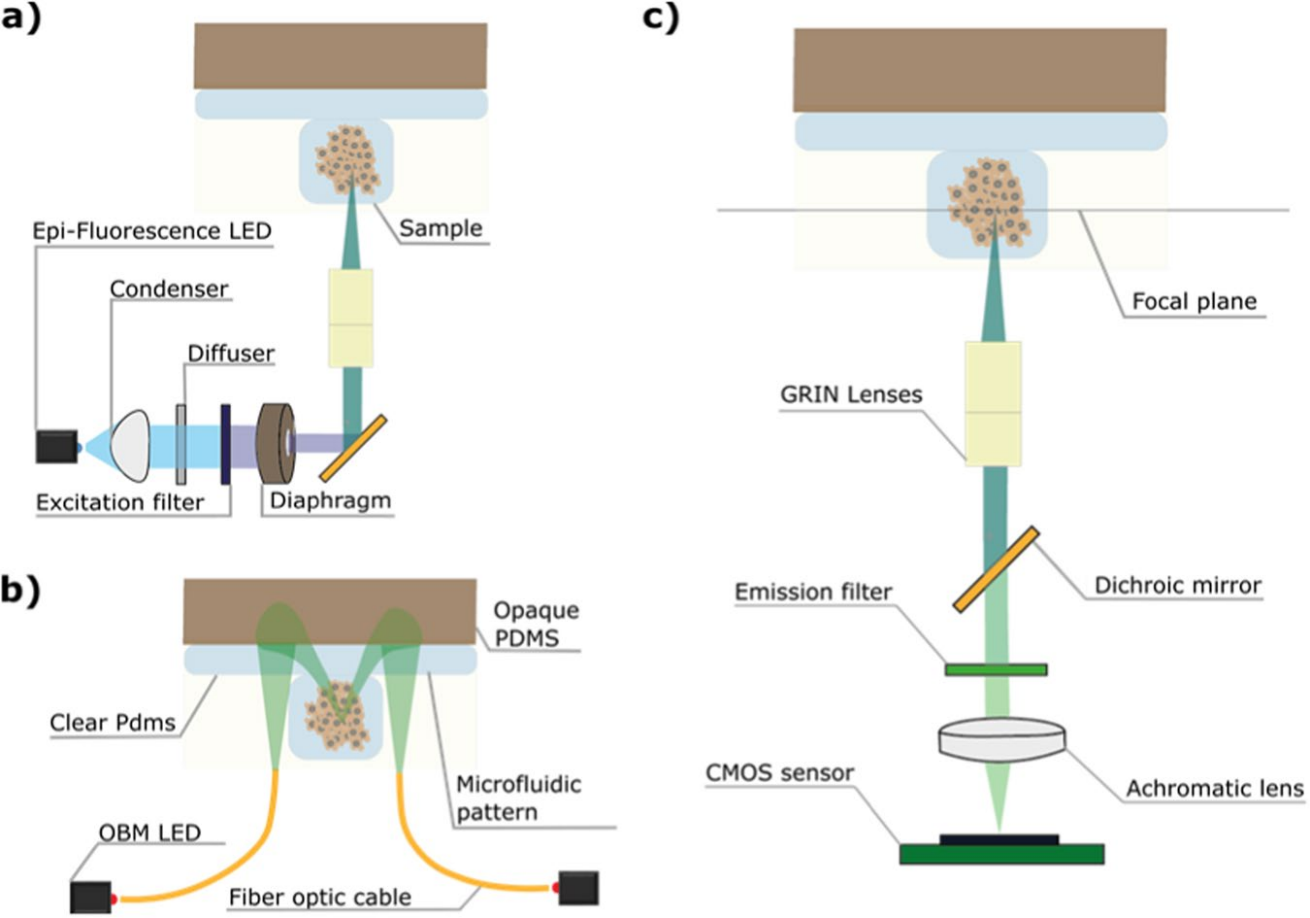
Schematic design of the illumination and image acquisition pathways of the Spheroscope. (**a**) Epi-illumination pathway. (**b**) OBM illumination pathway. (**c**) Image acquisition pathway.

The illumination and image acquisition paths of the system are shown in Fig. 2 and described in the following paragraphs:

#### Illumination

Epifluorescence illumination (Fig. 2a), is provided by a blue LXML-PB01-0040 LED (*Philips Lumileds*, San Jose, USA) attached to a heatsink. A condenser lens ACL12708U-A, *f* = 8 mm, (*Thorlabs*, Newton, USA) collimates the light through a diffuser-lens DG05-600 (*Thorlabs*, Newton, USA), an excitation filter BP470/35 (*Delta Optical*, Hørsholm, Denmark) and a 4.25 mm diaphragm that blocks off-axis rays. A dichroic mirror FF495-Di03 (*Semrock*, Rochester, USA) placed at 45 degrees relative to the direction of the light, reflects the rays towards the objective lens, which focuses the light rays onto the sample. For OBM illumination (Fig. 2b), two green M530F2 LEDs (*Thorlabs*, Newton, USA) are used, each coupled to a fibre optic cable FT400EMT-custom (*Thorlabs*, Newton, USA) that guides the light to the sample, at both sides of the objective lens, with adjustable distance and angle of incidence.

#### Image acquisition

The objective lens is composed of two vertically stacked GRIN lenses. The first lens, GT-IFRL-200-001-50-NC (*Grintech*, Jena, Germany), collects the light rays coming from the sample, located at the lens’ focal plane, 1 mm from the front surface, and creates an image of the sample at the lens’ rear surface. The second lens, GT-IFRL-200-inf-50-NC (*Grintech*, Jena, Germany), with a working distance of 0 mm, collects the image from the rear surface of the first lens and projects it to infinity as a set of parallel rays. These at-infinity rays travel through the dichroic mirror and an emission filter FF01-525/45 (*Semrock*, Rochester, USA). An achromatic doublet lens SN49-759, *f* = 19.1 mm (*Edmund Optics*, New Jersey, USA) collects and focuses these rays onto the microscope CMOS sensor daA2500-14 um bare model (*Basler*, Ahrensburg, Germany) (Fig. 2c). The sensor is controlled using the *Basler* default software *Pylon Viewer* through a USB 3.0 interface. The resolution, magnification, field of view (FOV) and working distance (WD) of the Spheroscope is determined by the first GRIN lens and the achromatic lens, as well as by the gap between the achromatic lens and the CMOS sensor, which can be adjusted through a placement wheel, thus allowing the use of achromatic lenses with different focal lengths.

The Spheroscope CMOS sensor has 5 million 2.2 × 2.2 μm pixels, packed in an area of 5.7 × 4.2 mm^2^. The images acquired are 2592 × 1944 pixels, 14 bits deep. Images acquired on fluorescence mode do not require postprocessing.

**Figure 3:**
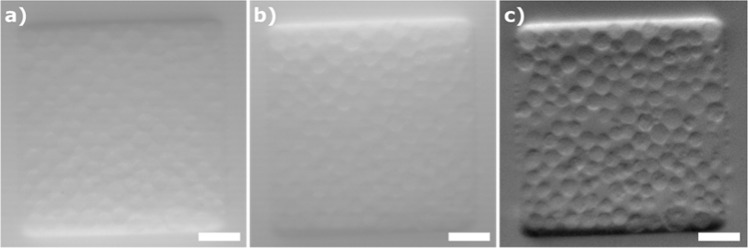
OBM image reconstruction. H1299 cells loaded in a microfluidic device. (**a**) Raw image obtained illuminating with the right LED (*I*_*right*_). (**b**) Raw image obtained illuminating with the left LED (*I*_*left*_). (**c**) Reconstructed OBM image. The size bar indicates 50 μm.

Phase-gradient contrast imaging builds on the principles of oblique back illumination microscopy (OBM), introduced by Ford *et al*. [2012]^20^. To this end, two images ${I}_{left}\,\& \,{I}_{right}$ are acquired, alternatively illuminating the sample with two diametrically opposed off-axis light sources. These images are then combined to generate a phase-gradient contrast, *I*_*OBM*_ image^18,21^:

1 $${I}_{OBM}=\frac{1}{2}\ast (\frac{{I}_{left}}{{I}_{{G}_{left}}}-\frac{{I}_{right}}{{I}_{{G}_{right}}})$$

where ${I}_{{G}_{left}}\,\& \,{I}_{{G}_{right}}$ are Gaussian (σ = 100 pixels) filtered version of ${I}_{left}\,\& \,{I}_{right}$, respectively. The ½ factor is used, as described by Ford & Mertz [2013]^21^, to give the final phase-gradient contrast image units of percentage deviation from the background. A median filter (kernel size of 3 × 3) is applied to remove noise in *I*_*OBM*_. Negative values are dealt with by offsetting all pixel values with the most negative pixel value of the image. For display purposes, images are contrast stretched by grayscale mapping from 0 -black- to 255 -white-. An example is shown in Fig. 3.

#### Characterization

The Spheroscope was characterized both under OBM and epifluorescence illumination modes. The magnification and effective field of view, which are independent of the modality, were measured under OBM illumination using calibration slides. Please note that, since the calibration slides displayed printed structures with no depth information, phase-gradient contrast images display very little or no information. Therefore, the images of the calibration slides were obtained using the absorption information instead of the phase provided by the OBM illumination, by replacing the subtraction sign in Eq. 1 with an addition sign. The resolution was calculated from the numerical aperture of the GRIN lenses. The sensitivity of the detection was measured on epifluorescence images of slides containing calibration beads. As final validation of the system in both modalities, the accuracy of the detection of 20 μm PMMA fluorescence beads 105-70-020 (*PolyAn*, Berlin, Germany) imaged both in phase-gradient contrast and fluorescence mode was calculated.

**Figure 4:**
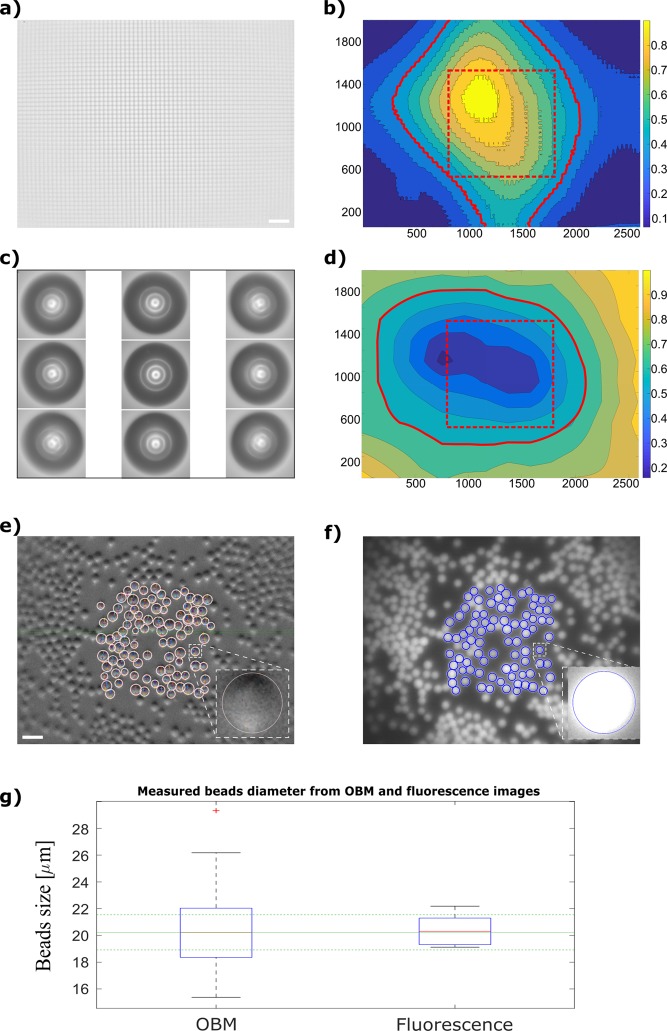
Spheroscope characterization. (**a**) 10μm grid used to calculate the magnification; (**b**) Flat field map computed from the local variance of the grid in (**a**). The continous red line represetnts the flat field area and the red dashed square shows the effective FOV; (**c**) Image of the circle pattern used to calculate the aberration free area, centred at nine positions of the image field, namely from left to right and top to bottom: (345,1614); (1374,1614); (2268,1614); (345,1039); (1374,1039); (2268,1039); (345,264); (1374,264); (2268,264) pixel coordinates; (**d**) Aberration map computed from the eccentricity of the pattern of concentric circles shown in (**b**), located at periodically equidistant locations of the entire image field. The continous red line represetnts the free aberration area and the red dashed square shows the effective FOV; (**e**) Spheroscope’s phase-gradient contrast image of a spread of 20μm calibration beads, displaying the segmentation outlines of the beads located inside the effective field of view; (**f**) Spheroscope’s fluorescence image of a spread of 20μm calibration beads displaying the segmentation of the beads contained within the effective field of view of the Spheroscope; (**g**) Mean bead diameter measured from the OBM and fluorescence images. Horizontal lines represent, as a reference, the mean (solid green line) and standard deviation (dashed green lines) diameter values provided by the manufacturer. Note: the size bar indicates 50μm.

The magnification of the system was measured using the 10 μm-spacing calibration grid shown in Fig. 4a. Ten (10) central, consecutive grid boxes, amounting to a distance of 100 μm of the grid, occupied 404 pixels of the image of the grid captured by the CMOS sensor. This corresponds, given the sensor’s pixel size (2.2 μm), to a projected length of 888 μm. Therefore, the magnification of the Spheroscope is 888 μm/100 μm = **8.88X**.

The effective field of view (FOV) of the Spheroscope was derived from the calculation of the flat field and aberration field of the system. On the one hand, using the 10 μm-spacing calibration grid shown in Fig. 4a, the field curvature was measured as the degree of focusing of the grid lines across the image, which in turn was calculated as the local variance^22^ (Eq. 2):

2 $${F}_{var}=\frac{1}{MN}\mathop{\sum }\limits_{i=1}^{M}\mathop{\sum }\limits_{j=1}^{N}{(u(i,j)-\bar{u})}^{2}$$

where M and N are the length and width, in pixels, of the window where the variance is computed, (in our case 200 × 200), *u*(*i*, *j*) is the intensity of the pixel with coordinates *i, j* of the window, and *ū* is the mean intensity of the pixels of the window. The field curvature map obtained, by calculating the variance in 200 × 200 windows in all the pixels of the image in Fig. 4a is shown in Fig. 4b.

On the other hand, using the calibration slide containing the concentric circle pattern shown in Fig. 4c, an aberration map was computed from the eccentricity of the concentric circle pattern, centred in locations uniformly distributed across the field of view. This was done by acquiring 88 images of shifted versions of the concentric circle pattern, centred every 52 μm in both, x and y directions, thus forming a grid of 8 rows and 11 columns. These images were combined into a single image. Figure 4c shows a partial reconstruction of the image displaying the concentric circle pattern as seen in nine representative location within the area imaged by the sensor. The outer circle of the pattern was used to calculate the eccentricity, after being segmented from the image array using a sequence of image processing *Fiji*^23^ plugins: first the “Rolling Ball Background Subtraction” plugin^24^, was applied with a 20-pixel radius structuring element. Then, the circle was segmented from the images using the “Otsu” thresholding algorithm^25^. The resulting binary mask was median-filtered with kernel size of 3 × 3 to remove noise. The eccentricity of the circles was then measured from the segmented area. To obtain a continuum aberration map, linear interpolation was applied to the discrete values calculated. The aberration map obtanied is shown in Fig. 4d.

The effective field of view of the Spheroscope was estimated from the intersection between the flat area, determined from the map shown in Fig. 4b and the aberration-free area, measured from the map shown in Fig. 4d. To this end the flat area was defined as the area of the map where the deviation of the variance was below 50% and the aberration-free area was defined as the area of the map where deviation of the eccentricity from a perfect circle was below 30%. This intersection encloses what we define as the effective FOV, consisting in a square of 1024 × 1024 pixels (the dashed red square in Fig. 4b,d). This usable aberration free FOV corresponds to **2.25 × 2.25 mm**^2^ of the sensor, or **254 × 254 μm**^2^ of the sample.

The lateral resolution of the Spheroscope was calculated using Abbe’s diffraction law^26^, as the ratio between the wavelength of the light used to image the sample (λ) and twice the numerical aperture of the objective (NA). In fluorescence mode, being the wavelength of the excitation LED 470 nm and the NA of the grin lenses 0.6, the theoretical lateral resolution is 470 nm/1,2 = **391 nm**. In OBM mode, being the wavelength of the excitation LED 530 nm and having the same NA, the theoretical lateral resolution is 530 nm/1,2 = **441 nm**.

The sensitivity of the Spheroscope in fluorescence mode was measured using the *Sphero GFP* Calibration Particle Kit. This kit contains 3.0−3.4 μm diameter fluorescent beads that are excited at 489 nm, and emit at 510 nm. These beads display six increasing levels of fluorescent intensity, referred to as PEAK levels 1–6, measured in normalized mean fluorescence intensity (MEF) units^27,28^. The MEF values for PEAK levels from 1 to 6 are 0.42 (PEAK 1), 2.751 (PEAK 2), 11.914 (PEAK 3), 130.96 (PEAK 4), 317.386 (PEAK 5), and 621.248 (PEAK 6). First, the evolution of the signal to noise ratio (SNR) provided by the Spheroscope was measured. To this end, for each PEAK level, 7 bead spreads were mounted on standard microscope slides. Then 7 experimental conditions were established, by varying the LED’s intensity and the exposure time. Namely, first the LED’s intensity was set at 50% of its maximum power and the exposure time was increased from 250 ms to 1000 ms in 250 ms steps. For the remaining three experimental conditions, the exposure time was fixed to 500 ms and the LED’s intensity was set to 25, 75 and 100%, respectively. Please note that the LED’s intensity percentages are defined by the driving current of the LED, adjustable between 0 and 1 A. According to the LED’s datasheet, 25%, 50%, 75% and 100% LED’s intensities correspond, respectively to 30.75 lm, 51.25 lm, 73.8 lm and 90.2 lm luminous flux values.

For each experimental condition and PEAK level, 7 images were acquired, one per slide. The images were processed as follows: first, background subtraction was applied using *Fiji*’s “Rolling Ball Background Subtraction” plugin to normalize the images and suppress background inhomogeneities. Then, the fluorescent signal was segmented by applying the “Otsu” thresholding method, followed by a median filter (kernel size of 3 × 3) to remove noise. This produced a mask of the image areas occupied by beads, which was then refined using a binary “Watershed”^29^, to separate touching beads and remove artefacts. A final size threshold was applied to remove too large (>450 pixels) or too small (<100 pixels) objects, thus leaving only binary masks corresponding to the isolated beads.

From the images processed as described above, the average and standard deviation of the beads and background intensity was then calculated. To calculate the intensity of the beads, the segmented bead masks were eroded using the “Erode” *Fiji* plugin with a 3 × 3 square structuring element. The beads’ intensity was then measured as the mean value of all pixels left. To calculate the intensity of the background, the segmented bead masks were dilated twice using the “Dilate” *Fiji* plugin with a 3 × 3 square element. The inverse of the dilated mask was considered background. The background intensity was measured as its average. Since *PolyAn* beads produce a non-uniform -Gaussian shaped- intensity profile, the noise cannot be properly computed from the signal masks, as the noise measurements would be contaminated by the signal variations. Therefore, we follow the widely accepted alterantive of computing the noise from uniform background areas. Accordingly, the signal-to-noise ratio is computed as: SNR = C/σ_B_, being C the average intensity of the beads and σ_B_ the standard deviation of the background^30,31^. SNR for each PEAK level was calculated in *Matlab*.

**Figure 5:**
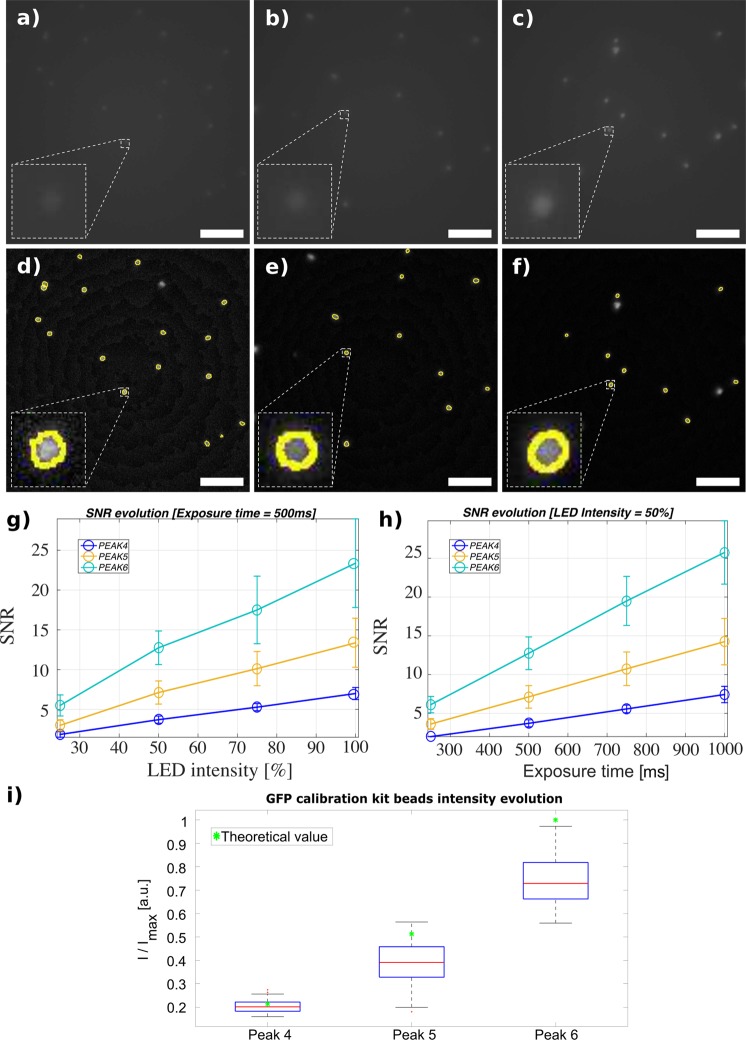
Sensitivity study using different normalized intensity beads. (**a**,**d**) PEAK 4, (**b**,**e**) PEAK 5 and (**c**,**f**) PEAK 6 bead images (exposure time:1000 ms; LED intensity: 50%). (**a**–**c**) Raw images. (**d**–**f**) Segmentation results. The size bar indicates 50 μm. (**g**) SNR evolution as a function of LED intensity (exposure time:500 ms). (**h**) SNR evolution as a function of the exposure time (LED intensity: 50%). (**i**) PEAK 4, PEAK 5, and PEAK 6 beads measured intensities from images obtained with the Spheroscope. The green asterisk represents the theoretical intensity values of the different PEAKs.

The evolution of the SNR, for each PEAK level, as a function of the percentage of the maximum intensity provided by the LED (L_p_) and the exposure time (t_exp_) is shown in Fig. 5i,j. The analysis revealed a lower detection limit at PEAK3 level. Otherwise, following the Albert Rose criteria, according to which an image can be segmented if it has a SNR value above 5^31,32^ it can be concluded that, for a reasonably low exposure time of 500 ms, detection of PEAK4 requires using the LED at 75% intensity, PEAK5 microspheres can be properly detected above 38% LED intensity and PEAK6 microspheres are detectable even at 20% LED intensity. Similarly, working at 50% peak LED intensity, microspheres are detectable above 700, 350 and 200 ms for PEAK4, PEAK5 and PEAK6 respectively.

Then the relative intensities between PEAK levels were measured to determine if the Spheroscope is able to reproduce the theoretical differences reported by the manufacturer. To this end, beads from PEAK levels 4, 5 and 6 were spread on standard microscopy glass slides, and eight images were acquired from each slide (Fig. 5). From these images, processed as it has been described in the previous paragraphs, the bead median and quartile intensity distribution was computed for each PEAK group level (Fig. 5g). The results reveal that the intensity levels follow the theoretical values (green asterisk in Fig. 5g).

In summary, the detection of the fluorescent beads above PEAK3 is feasible at a large range of intensity and exposure time values. The exposure time and laser intensity for a particular application must be set within those ranges, balancing sometimes opposing requirements, such as the time available for the acquisition, the photobleaching of the fluorochrome, or the phototoxicity that the light might inflict to the cells.

Finally, the quality of the images was evaluated, both in fluorescence and phase-gradient (OBM) modes, by estimating the size of calibration beads from Spheroscope images (Fig. 4e,f). To this end, 20 μm PMMA fluorescence beads 105-70-020 (*PolyAn*, Ahrensburg, Germany) were used that are excited between 450–565 nm and emit between 540 and 620 nm. The beads were spread on a standard microscope slide. Five images were taken using the Spheroscope in both fluorescence and phase-gradient mode. Then the beads located inside the effective FOV of the microscope were segmented as described in the following paragraphs.

In fluorescence mode, a contrast-limited adaptive histogram equalization (CLAHE) was applied to the images, followed by *Matlab*’s “imfindcircles” function, limited to the effective FOV, which retrieved all the beads located in the FOV. To this end, it maximizes the contrast of the Hough transform of the image, for a given range of radii values. In phase-gradient contrast images, a home-made algorithm was used. All beads contain an area of dark pixels and white pixels located at two diametrically opposed areas of the bead’s edge (Fig. 4e). Therefore, from a pixel located at the bead’s center, the algorithm radially looks for the darkest (p_1_) and brightest (p_2_) pixel areas, the distance between p_1_ and p_2_ corresponding to the sphere diameter. A total of 348 beads were processed as described.

Examples of the segmentation, both in phase-gradient contrast and fluorescence are shown in Fig. 4e,f. The comparison of the measurements obtained using both Spheroscope’s modes is shown in Fig. 4g. As shown, the average bead diameter measured on Spheroscope’s phase-gradient contrast images (20.21 ± 2.54 μm) is non statistically different from the average diameter measured on fluorescence images (20.34 ± 1.13 μm). Both values are also comparable with the values provided by the manufacturer (20.23 ± 1.31 μm).

### Experimental results

#### Device design and fabrication

**Figure 6:**
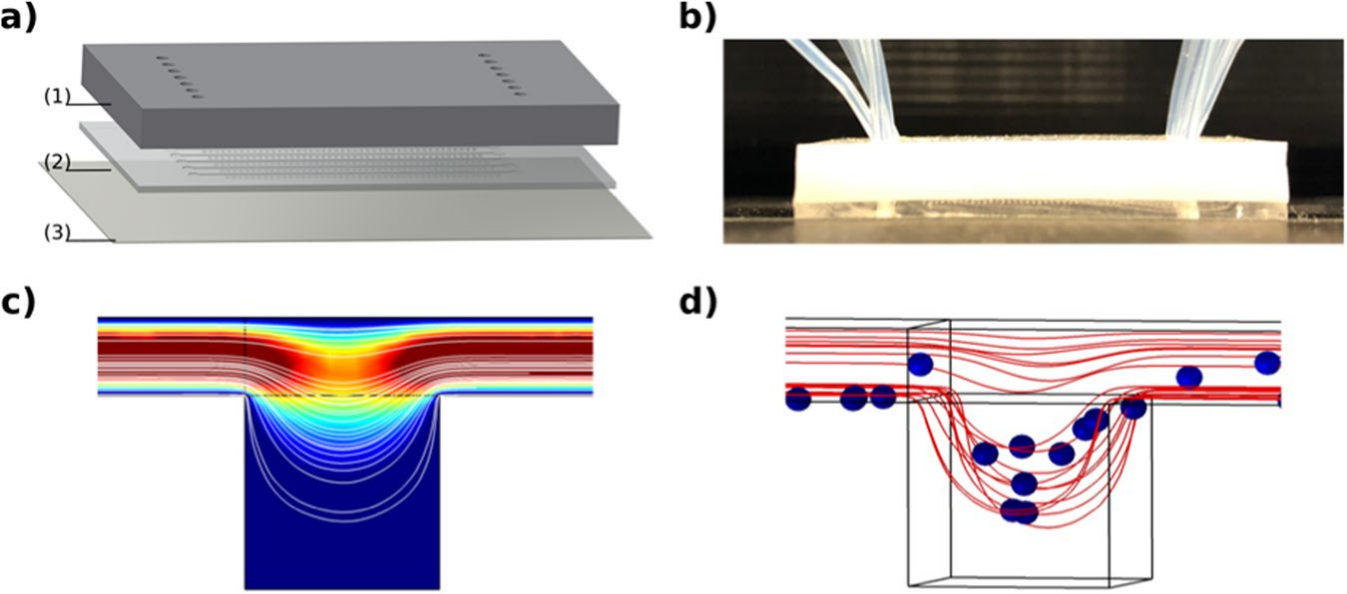
Microfluidic device and flow simulation. (**a**) Sketch of the microfluidic device. (**b**) Fabricated microfluidic device. (**c**) Simulated velocity profile of the fluid flow and streamlines entering in one single microdevice chamber at 0.5 μl/min flow rate. (**d**) 3D simulation of the trajectories of 10μm diameter particles, at 0.5 μl/min flow rate.

The microfluidic device consists of three layers (Fig. 6a). The top and middle layers are made of Polydimethylsiloxane (PDMS). The bottom layer is a commercial glass cover-slip that provides rigidity and facilitates handling the device during the experiments. The top layer covers the microfluidic channels, and is also patterned with the inlets and outlets of the device. The central layer, which contains the microfluidic pattern, was designed with 7 rows of 42 250 × 250 × 250 μm cubic wells connected by a 250 × 100 μm channel located on top of the wells. Figure 6b shows an image of one of the microfluidic devices, with the loading tubes connected.

#### Loading of the device

Cells embedded in culture medium are inserted through the inlets of the microdevice. Based on simulations performed (Fig. 6c,d), at 0.5 μl/min loading flow, the velocity of the cells is slow enough to ensure that the cells are seeded by gravity into the micro-wells. Once the cells are captured in the wells, the anti-attachment surface treatment used prevents the cells from attaching to the walls or the bottom of the well. Therefore, the cells, missing a suitable substrate, attach to each other and form spheroids.

#### Experiment 1: Comparison of Spheroid size

**Figure 7:**
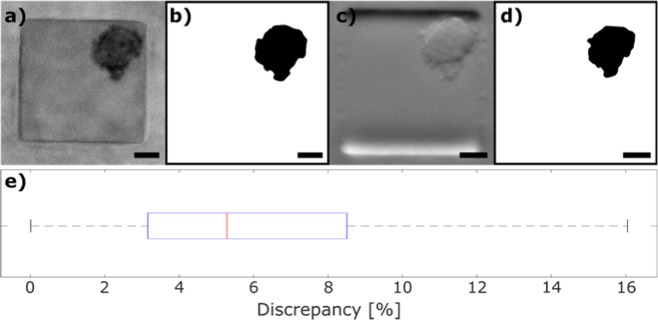
Comparison between microscopes. (**a**,**b**) Manual segmentation of a spheroid from an image obtained using a *Zeiss Cell Observer* microscope, with an N-Achroplan 10 × 0.2NA objective and an Axiocam MRm camera, (**c**,**d**) Manual segmentation of the same spheroid from an image obtained using the Spheroscope. (**e**) Boxplot showing the discrepancy between the manual segmentation of 113 spheroids imaged using both systems. The size bar indicates 50 μm.

To compare the quality of the Spheroscope with that of a commercial microscope, 113 spheroids were created in the microfluidic device and imaged both with the Spheroscope in phase-gradient contrast mode and with a commercial *Zeiss Cell Observer* microscope in phase contrast mode, using a N-Achroplan 10 × 0.2NA objective and an Axiocam MRm. Namely, murine cells from a pancreatic ductal adenocarcinoma cell line PDAC93 KRAS P53^H^ PTF1-CREKI were loaded into the microfluidic device using the protocol described above, and kept in an incubator during 48 hours, until the spheroids were formed. Then the same 113 spheroids were imaged using both microscopes and manually segmented using a custom *Fiji* macro. The area was measured from the segmented masks. The discrepancy between systems was defined, for each spheroid, as the ratio between the smaller and the bigger segmented area. Our results show that the mean discrepancy between segmented areas is 5% (Fig. 7).

#### Experiment 2: Formation and follow-up of tumour spheroids

**Figure 8:**
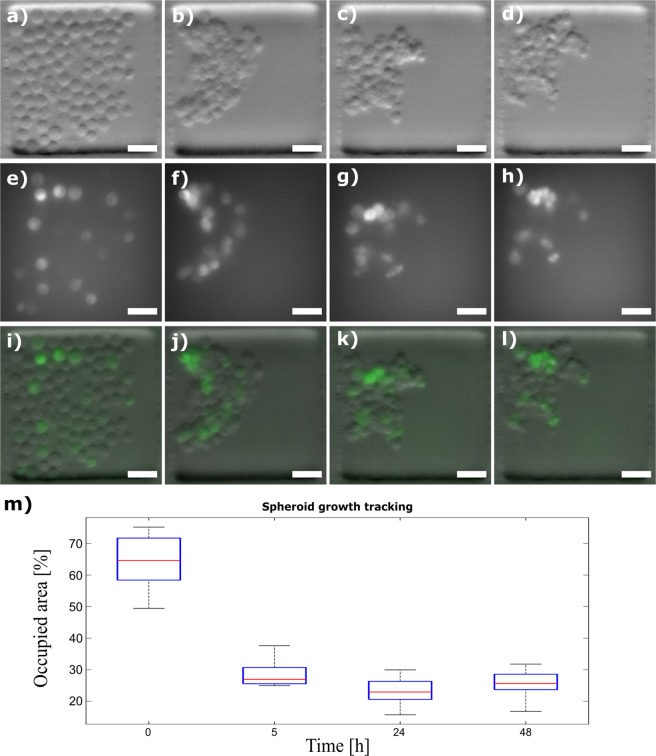
Spheroscope images of the evolution of a spheroid during its first 48 hours. (**a**–**d**) show the images obtained with OBM illumination, (**e**–**h**) show the images obtained with fluorescence, (**i**,**j**,**k**,**h**) show the combination of both images at 0 (**a**,**e**,**i**), 5 (**b**,**f**,**j**), 24 (**c**,**g**,**k**) and 48 hours (**d**,**h**,**l**) and (**m**) shows the tendency of how cells are grouped. Size bar indicates 50 μm.

To determine the ability of the Spheroscope to monitor the tumour spheroid formation process, it was used to track the formation of spheroids created from non-small lung cancer cell line H1299-GFP. Namely, H1299-GFP lung cancer cells were loaded into the microfluidic device using the protocol described above. Fluorescence and phase-gradient OBM images of fourteen (14) samples were acquired in time lapse mode 0, 5, 24 and 48 hours after loading. Figure 8 shows the formation and evolution of spheroids both in OBM (a-d) and fluorescence (e-h). The evolution of the spheroid formation was evaluated by measuring the area occupied by the spheroid, manually segmented from the images using a custom macro in *Fiji*. Figure 8m shows that the area occupied by the loaded cells quickly decreases during the first hours of the experiment (from 0 to 5 hours). At this point, the cells assemble and the spheroids are formed. Afterwards, the spheroid remains with a similar size.

**Figure 9:**
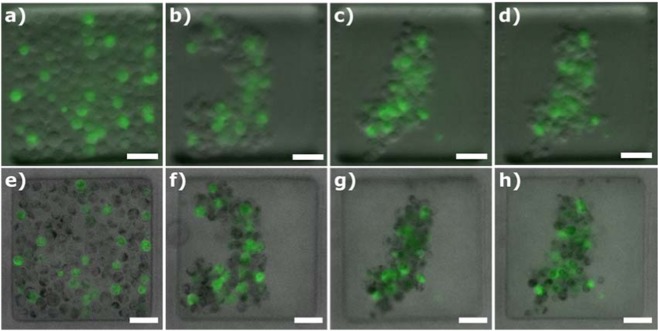
Comparative of the Spheroscope and a *Zeiss Cell Observer* microscope, with an N-Achroplan 10 × 0.2NA objective and an Axiocam MRm camera. The sequences show the same spheroid at 0 (**a**,**e**), 5 (**b**,**f**), 24 (**c**,**g**) and 48 hours (**d**,**h**) acquired with the Spheroscope -fusion of OBM and epi-fluorescence images- (**a**–**d**) and with the *Zeiss Cell Observer* microscope – fusion of transillumination brightfield and epi-fluorescence images- (**e**–**h**). The size bar indicates 50 μm.

Finally, a comparison was performed between images of the spheroids, obtained at end-time, using both the Spheroscope and the commercial *Zeiss Cell Observer* microscope. Figure 9 shows both series of images. Based on the obtained data, we conclude that the Spheroscope parallels the quality of the commercial microscope at this level of resolution, both in fluorescence and brightfield mode.

## Discussion

High-throughput *in vitro* analysis is becoming mainstream in the process of validating the efficacy of anti-cancer drugs. This has been traditionally done on standard 2D cultures, which do not physiologically resemble the situation of cancer cells *in vivo*. Alternatively, novel 3D cell models are increasingly being used that provide a more physiological environment for these studies. Therefore, the effect of a drug measured in those models is more representative of the possible efficacy of the drug for the patient. In particular, tumour spheroids, formed from clusters of cells in suspension, constitute primordial structures where anticancer drugs are more efficiently studied than in standard 2D cultured cells.

The analysis of the effect of the drug on a spheroid, measured using a simple metric such as the growth rate of the spheroid, is commonly calculated using complex, expensive equipment, enabled with high quality optics and control electronics. Furthermore, these studies are usually done on multi-well plates of relatively large dimensions and a fixed structure, with the corresponding cost in reagents and little experimental flexibility.

Here we have presented the Spheroscope, a custom-made low resolution brightfield and fluorescence microscope, built following the DIY philosophy. We show how with its low-cost components, the Spheroscope can successfully image the formation and growth of 3D spheroids created from isolated tumour lung cancer cells and grown in high-throughput mode inside a customized microfluidic platform. We have described the Spheroscope prototype conception and fabrication. Also, we have characterized the microscope prototype sensitivity: effective FOV, magnification, resolution and sensibility, all of which have been found sufficient for the task of efficiently imaging tumour spheroids and study their development and viability up to 48 hours.

Future work will be to industrialize the system to allow for the automation of the stage and the creation of an incubated environment were high-throughput follow up of spheroid growth can be monitored in a large number of wells. This industrial device will be complemented with control software and integrated basic image analysis tools.

## Materials and Methods

### Cells and cell culture

The non-small lung cancer cell line H1299 was obtained from the ATCC (*American Type Culture Collection*). Cells were stably transfected with a vector that expresses the GFP reporter protein pEGFP-C1 (*Clontech*, Mountain View, USA). The pancreatic ductal adenocarcinoma cell line PDAC93 KRAS P53^H^ PTF1-CREKI was obtained from a pancreatic tumor developed in a murine model. The PDAC93 cell line was donated by Dr. Silvestre Vicent (Center for applied medical research, CIMA).

H1299-GFP cells were cultured in a mixture of culture RPMI 1640 medium supplemented with 10% fetal bovine serum and 1% antibiotic (penicillin-streptomycin solution). Pancreatic cells were cultured with the same mixture, replacing RPMI 1640 with DMEM 1X. Cells were incubated at 37 °C and 5% CO2, and subcultured every forty-eight hours in culture flasks to maintain a conductive environment for cell proliferation. Before each experiment, cell concentration was calculated by Neubauer-Fischer test where typical concentration was 1.000.000 cells/ml.

### Microfluidic devices

PDMS layers are fabricated by standard replica moulding methods, using two different moulds. The middle (patterning) layer mould was made in a silicon wafer with positive relief SU8 structures created by conventional photolithography. The top layer mould was fabricated in a 3D SLA Form2 printer (*Formlabs*, Somerville, USA). To fabricate these PDMS layers, a mixture of Sylgard 184 PDMS (*Dow Corning Co*., Midland, USA) with a curing agent (1:10 v/v curing agent to base ratio) was poured in the moulds. The mix was degassed under vacuum for 30 minutes. White acrylic paint was introduced in the PDMS mixture step (1:50 v/v acrylic paint to PDMS mixture) to make the top layer opaque, as required for OBM imaging. The PDMS is cured at 70 °C for four hours.

Due to the small working distance of the Spheroscope (1 mm), it was necessary to minimize the thickness of the bottom PDMS layer. To this end, two structures of 500 um were bonded around the silicon wafer to act as top end. After pouring and degassing the PDMS, a flat glass surface treated with sigmacote (*Sigma*, St. Louis, USA) was placed over the uncured PDMS, in contact with the 500 um structures, to produce the 500 um width PDMS layers.

After unmolding, the two PDMS layers were permanently bonded to each other by oxygen plasma treatment at 40 W during thirty seconds using a Zepto (*Electronic Diener*, Ebhausen, Germany) plasma system. The same protocol was repeated to bond the glass cover-slip with the PDMS set. Finally, to enhance bonding, the microfluidic devices were introduced inside an oven at 70 C for more than one hour.

### Surface treatment

Twenty-four hours before loading the cells, a surface treatment is required to prevent cell attachment to the inner surfaces of the wells and channels. To this end, 1% (v/v) Synperonic F-108 (*Fluka*, Sigma-Aldrich, Co., St. Louis, USA) was manually introduced into the channels through the inlets with a syringe connected to a 24-gauge PTFE tube through a 22-gauge cannula. Synperonic was left in the device for at least 4 hours. During this time, the device was UV sterilized at least for two hours (*Stratalinker* UV Crosslinker). Before loading the cells mixed with medium, Symperonic was removed from the channels by flowing mounting medium from the inlets.

### Spheroid formation and imaging

Once the device was ready, cells were introduced at a concentration of one million cells per millilitre using a syringe pump. To determine the optimum flow rate, simulations were performed using *COMSOL* Multiphysics (*COMSOL* Inc., Stockholm, Sweden) to choose the initial range of fluid working flows. The main objective of the simulations was to define a range of velocities where the gravitational force is large enough to seed in the chambers cells that are located in the lower streamlines of the fluid. *COMSOL* simulation allows to verifies the seeding and presence of cells in all wells along the main channel. Once cells were seeded in the wells, two images per chamber were obtained during the spheroid formation at 0, 5, 24 and 48 hours at an exposure time of 500 ms for phase-gradient contrast images and a 50% LED intensity and 500 ms of exposure time for fluorescence images. The same capture times were repeated for images taken with a commercial epifluorescence microscope (*Zeiss Cell Observer*).
